# A spike in mechanotransductive adenosine triphosphate release from red blood cells in microfluidic constrictions only occurs with rare donors

**DOI:** 10.1111/micc.12439

**Published:** 2018-04-11

**Authors:** Jordan E. Mancuso, William D. Ristenpart

**Affiliations:** ^1^ Department of Chemical Engineering University of California Davis Davis CA USA

**Keywords:** adenosine triphosphate, erythrocytes, mechanotransduction, microfluidics

## Abstract

**Objective:**

Wan et al (*Proc Natl Acad Sci USA*,** 105**, 2008, 16432) demonstrated that RBCs rapidly and transiently release a spike of 300% more ATP shortly downstream from a short microfluidic constriction where the cells experience a sudden increase in shear stress. More recent work by Cinar et al (*Proc Natl Acad Sci USA,* **112**, 2015, 11783), however, yielded no evidence for a similar spike in ATP release downstream of the constriction. Our aim was to determine whether a transient spike in mechanotransduction is the typical response of RBCs to the sudden onset of increased shear.

**Methods:**

We investigate ATP release downstream of a microfluidic constriction for 15 participants using a luciferase‐based photoluminescent assay.

**Results:**

While we observe mechanotransductive ATP release from blood drawn from all donors, we find evidence of a spike in ATP concentration after the microfluidic constriction for only 2 of 15 participants. No clear trends in ATP release are found with respect to the magnitude of the applied shear stress, or to the gender, age, or physical activity (Baecke) index of the donor.

**Conclusions:**

In aggregate, all data acquired to date suggest that a spike in mechanotransductive ATP due to a suddenly applied increase in shear stress occurs in blood drawn from only 14% of the population.

**Abbreviations**
ATP: Adenosine triphosphate
BI: Baecke Index
Hct: Hematocrit
PDMS: polydimethylsiloxane
PPS: Photons per second
PSS: Physiological salt solution
RBC: Red blood cell

## 1 INTRODUCTION

Despite the traditional view of RBCs as passive carriers of oxygen, recent work has demonstrated that RBCs are active sensors of local circulatory conditions so that they can perform an important role in regulating local blood flow in the microvasculature.^1^, ^2^, ^3^ Specifically, RBCs have been shown to release ATP in response to hypoxia,^1^, ^4^, ^5^, ^6^ hypercapnia,^4^ mechanical deformation,^7^, ^8^, ^9^, ^10^, ^11^, ^12^, ^13^ changes in pH,^1^ and changes in osmotic pressure.^14^ When ATP is released from the RBC, it binds to purinergic receptors on nearby vascular endothelial cells.^15^, ^16^ This binding induces the synthesis and release of nitric oxide,^2^, ^6^, ^17^ which in turn leads to the relaxation of surrounding smooth muscle cells causing vasodilation.^18^ This vasodilation increases local blood flow, bringing more oxygen to areas in need.^19^

Changes in the local flow rate, and corresponding shear stress, are well known to cause RBCs to deform. Deformation‐induced release of ATP has been shown to be a major contributor to extracellular ATP,^2^ and many diseases have been linked to decreased ATP release such as cystic fibrosis,^20^ pulmonary hypertension,^21^ and diabetes.^22^ Early studies by Sprague et al^2^ investigated deformation‐induced release of ATP using filter paper with varied pore diameter. They found that as the pore diameter decreased, the amount of ATP released by RBCs increased. Subsequent studies by Sprung et al^8^ and Edwards et al^7^ corroborated this result using microbore tubing with inner diameters of 25‐75 μm.^7^, ^8^ This work improved on the filter paper experiments by allowing for collection of ATP release in a continuous flow system, but did not explore geometries consistent with those in the microvasculature.

The advent of microfluidics allowed for an investigation of ATP release in more physiologically relevant geometries. The earliest work investigating ATP release by RBCs in straight microfluidic channels was performed by Price et al,^9^ who found that decreasing cross‐sectional area and increasing Hct both led to increased ATP release. Later work by the same group^10^ utilized a channel that narrowed uniformly along the flow direction. They found that detected ATP in these channels was not governed by the smallest dimension of the channel, but was instead dependent on overall cross‐sectional area. Moehlenbrock et al^11^ improved on both designs using hydrodynamic focusing to investigate different cross‐sectional areas without physically changing the placement or geometry of the microchannel. This approach increased reproducibility, and again indicated that a decrease in cross‐sectional area increased detected ATP.^11^

Subsequent work by Wan et al^12^ focused on probing the dynamics of ATP release, using a microfluidic channel with a constriction that served as a mechanical stimulus at a prescribed location. Given the time‐invariant flow rate, measurements of ATP concentration as a function of position in the channel (using a standard luciferase‐based photoluminescent assay) allowed quantitative estimates of the time required for mechanotransduction to occur. They found that the ATP concentration in the channel remained low upstream of the constriction, reached a sharp peak following a delay after the onset of increased shear stress, and then decreased back to levels consistent with ATP concentration prior to the constriction. The magnitude of this spike varied with the length and width of the constriction and had a maximum value of 1.57 ± 0.17 μmol/L for a channel that was 800 μm long and 20 μm wide. The peak value for this channel, found at *x *≈* *1800 μm (measured from the constriction entrance), was approximately 260% greater than the ATP concentration prior to the constriction. Although the results in this study were highly reproducible, with 65 distinct experimental trials, all of the RBCs were drawn from a single participant.

Later work by Cinar et al^13^ was performed to identify specific ion channels responsible for the shear‐induced mechanotransduction. They utilized the same microfluidic channel dimensions that had yielded the maximum ATP release observed by Wan et al,^12^ a constriction 800 μm long and 20 μm wide. Although Cinar et al^13^ were able to demonstrate that the Piezo‐1 channel was associated with overall increased ATP release, the RBCs in the experiments did not exhibit any pronounced ATP release dynamics. Specifically, their observed peak was 0.81 ± 0.17 μmol/L, only approximately 20% greater than the initial magnitude of ATP. The “spike” was also located further downstream from the constriction at *x *≈* *3300 μm; most importantly the magnitude of the peak was smaller than the surrounding error bars, raising the question of whether a spike in mechanotransductive ATP release had even occurred. Notably, Cinar et al^13^ report a sample size of n = 11 measurements from 6 participants. The change in magnitude of the peak, in conjunction with the increase in sample size, suggests that the dynamics of ATP release may be more complicated than originally thought.

The work of Cinar et al^13^ complicates our understanding of Wan et al's^12^ results. It is clear from the large body of results acquired in filter paper experiments,^2^, ^20^, ^21^ microbore tubing experiments,^7^, ^8^, ^23^ and various microfluidic geometries^9^, ^10^, ^11^ that shear‐induced ATP release by RBCs is a significant contributor to extracellular ATP. Those experiments all involve increased shear stress of relatively long duration compared to the short constriction employed by Wan et al^12^ and Cinar et al^13^ used exactly the same short constriction geometry as Wan et al,^12^ yet did not observe a large spike in ATP. It is unclear if the sharp mechanotransductive dynamics reported by Wan et al,^12^ observed in blood drawn from only a single subject, are reproducible in the larger population. Furthermore, doubt has been cast on the mechanism for mechanotransduction, with some evidence pointing toward hemolysis as the primary mechanism for ATP release.^24^ However, there is some question as to whether the use of 1‐ to 14‐day‐old cells in work investigating hemolysis has obfuscated other mechanisms of ATP release.^25^

The main goal of this work was to assess whether a large spike in ATP mechanotransduction occurs downstream of a short constriction, using blood drawn from a larger cohort of participants. We show that RBCs drawn from n = 15 different participants all exhibited shear‐induced ATP release, consistent with previous results by numerous researchers who employed a variety of different mechanical stimuli. We go on to address the more specific question of whether or not there is an observable spike in ATP concentration immediately after a very short mechanical stimulus of shear stress using a microfluidic constriction, similar to that reported by Wan et al.^12^ We find that for 13 of the 15 participants, the ATP concentration is spatially invariant along the entire length of the channel, that is, no mechanotransductive spike was observed. We find no evidence that the magnitude of the flow rate or the characteristics of the donors (age, gender, and physical activity index) has a statistically significant impact on the spike (or lack thereof) in ATP release. Taking this work in conjunction with the previous efforts of Wan et al^12^ and Cinar et al,^13^ it appears that a pronounced spike in ATP mechanotransduction in response to a suddenly applied shear increase only occurs in RBCs drawn from about 14% of the population.

## 2 MATERIALS AND METHODS

**Figure 1:**
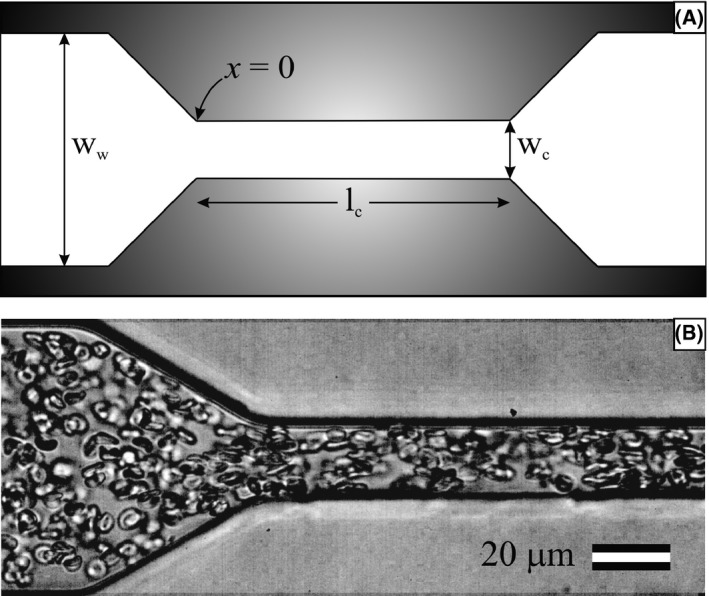
A, Schematic of the microfluidic constriction (not to scale). The microfluidic channel has a constriction from ww = 100 μm to wc = 20 μm. The length of the constriction is lc = 800 μm, after which the channel returns to ww = 100 μm. Note that *x* = 0 is defined at the beginning of the tapered region. B, Representative image of RBCs flowing through the constriction at *Q* = 2.5 μL/min and 10% Hct. Flow is from left to right

Microfluidic channels were fabricated with PDMS using soft photolithographic techniques by Duffy et al.^26^ The channels have a constriction from 100 to 20 μm with a taper angle of 60°. The height of the channel is 40 μm, and the length of the constriction is 800 μm. A schematic of the channel is shown in Figure 1A. We use this geometry to be consistent not only with Wan et al^12^ and Cinar et al^13^ but because it has been used extensively for investigations of the mechanical behavior^27^, ^28^, ^29^ of RBCs. The wall shear stresses for the wide region and constriction region are approximated as τ_*w*_ ≈ 6η*Q*/*h* ^2^ *w* and τ_*c*_ ≈ 6η*Q*/*w*_*c*_^2^ *h,* respectively, where η is the viscosity of the suspending fluid, *h* is the height of the channel, *w* is the width of the wide region, *w*_*c*_ is the width of the constriction region, and *Q* is the flow rate. This resulted in wall shear stresses of τ_*w*_ ≈ 0.16‐3.1 N/m^2^ and τ_*c*_ ≈ 1.6‐31 N/m^2^ for flow rates of 0.25‐5 μL/min. The corresponding shear rates are approximated as ${\dot{\gamma }}_{w}$ ≈ *Q*/*h* ^2^ *w *≈ 26‐520 s^−1^ and ${\dot{\gamma }}_{c}$ ≈ *Q*/*w*_*c*_^2^ *h *≈* *260‐5200 s^−1^ over the same range of flow rates.

**Figure 2:**
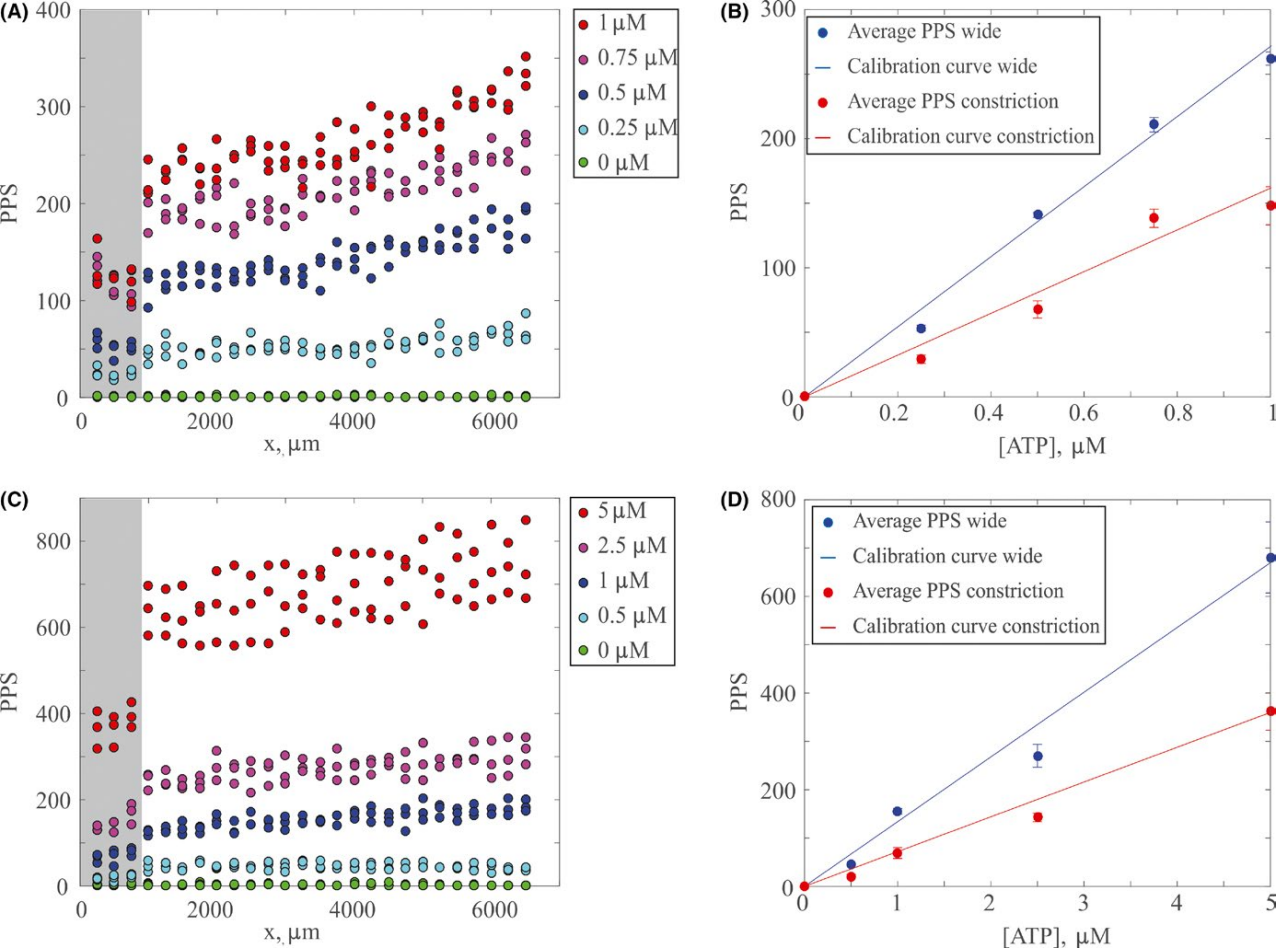
Representative ATP calibrations on separate days. (A) (C) PPS vs channel position, for varied ATP concentrations. Each color represents a different concentration of ATP, and each point represents a measurement of photons over 10 s cumulative at that position. (B) (D) Calibration curves for the wide region and constriction region. Each blue point represents the average PPS of all measurements for the wide region at that concentration of ATP, each red point represents the average PPS of all measurements of that concentration of ATP in the constriction. All error bars represent one standard deviation. Calibration data in (A) and (B) are taken from a different day than calibration data in (C) and (D), Luciferin/Luciferase solutions are slightly different on each day thus PPS for the same concentration of ATP may be slightly different. A distinct calibration curve was prepared for each day of experiments to account for differences in the enzyme activity to ensure an accurate calibration

ATP detection was performed using Luciferin/Luciferase in a fresh PSS (1.2 mmol/L MgSO_4_, 2.0 mmol/L CaCl_2_, 4.7 mmol/L KCl, 11.1 mmol/L dextrose with 1 mg/mL bovine serum albumin, 21.0 mmol/L tris(hydroxymethyl)aminomethane, and 140.5 mmol/L NaCl, pH adjusted to 7.4 with 10% HCl solution). To prepare the Luciferin/Luciferase solution, 300 μL of 1 mg/mL Luciferase extracted from *photinus pyralis* (Sigma) was added to a mixture of 5.1 mg D‐Luciferin (Sigma) in 15 mL of PSS. Calibrations were performed using concentrations of ATP from 0 to 1 μmol/L and 0 to 5 μmol/L, which were prepared using ATP sodium salt (Sigma) suspended in PSS. All solutions were prepared on the day of use. To ensure accurate calculation of ATP concentration and to confirm that the Luciferin/Luciferase system was functioning, calibrations were always performed on each experimental day using the same PSS, Luciferin/Luciferase, microfluidic channel, and photon counting system as for the RBC experiments (described below). Representative calibration curves are found in Figure 2; note that the spatial variation in PPS is due to the difference in cross‐sectional area between the constriction region and the wide region of the channel. When the cross‐sectional area exposed to the photomultiplier tube is larger, more light will be captured by the photomultiplier tube. Thus, two calibration curves are calculated from each scan along the channel, the first is for the constriction region, and the second is for the wide region of the channel (cf. Figure 2B,D).

**Figure 3:**
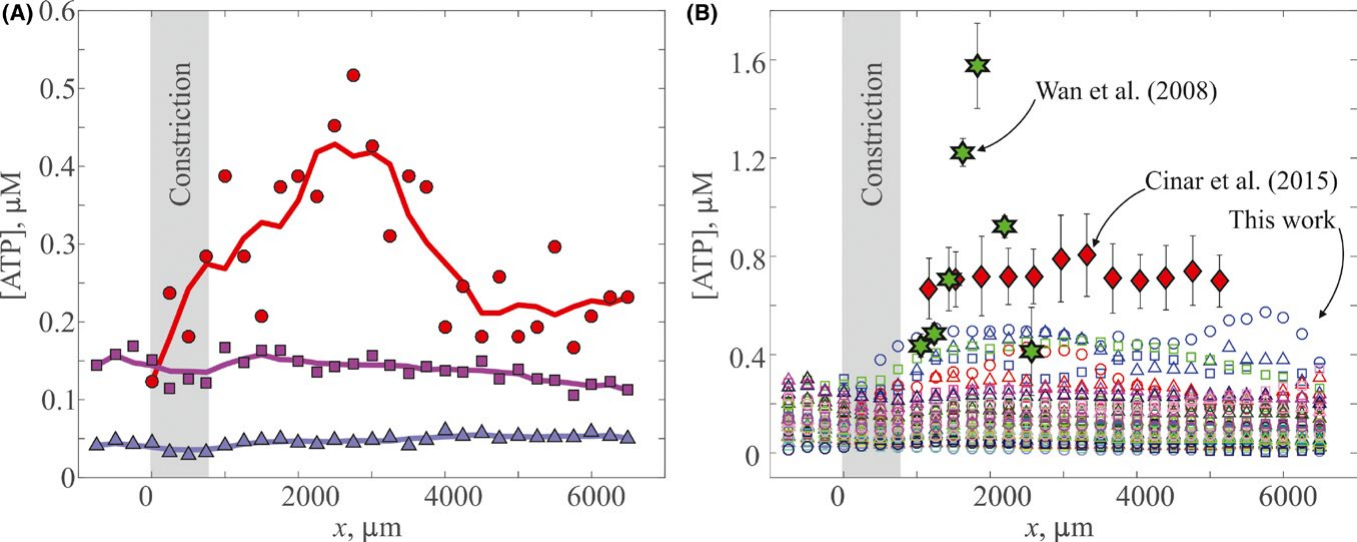
Comparison of detected ATP concentration vs downstream *x* position. (A) Representative trials from this work that typify the differences between participants who show a distinct mechanotransductive spike and participants who do not. Different colors denote individual participants, and separate shapes represent individual trials for a single participant. The solid lines represent the 5‐point running average. The shaded region on the figure corresponds to the constricted region of the microfluidic channel. (B) All trials from a flow rate of 2.5 μL/min are included here. The results from this work are displayed as single trials such that different colors denote individual participants, while separate shapes represent individual trials for a single participant. For this flow rate, we have n = 45 measurements from 15 different participants. Red diamonds represent the data extracted from the work of Cinar et al,^13^ with error bars showing the standard deviation of n = 11 measurements for 6 participants. Green stars represent the data extracted from the work of Wan et al,^12^ with error bars showing the standard deviation of n = 5 measurements for one participant. The shaded region on the figure corresponds to the constricted region of the microfluidic channel

Blood was collected from 15 participants under pre‐approved Institutional Review Board protocols for the study of ATP release by RBCs. A tourniquet was used during blood collection, and whole blood was collected into tubes containing heparin to prevent coagulation. To isolate RBCs, whole blood was centrifuged at 500 g at 20°C for 1 minute. The supernatant and buffy coat layers were aspirated off, and packed RBCs were resuspended and washed three times in PSS. These centrifugation parameters are consistent with, or gentler than, those used in previous studies investigating ATP release by RBCs^2^, ^7^, ^8^, ^9^, ^10^, ^11^, ^12^, ^23^ to reduce exposure of RBCs to shear stress prior to microfluidic experiments. These centrifugation parameters produce less than 0.01 μmol/L of ATP as measured in the packed RBCs (ie, over an order of magnitude less than ATP measured in this system, cf. Figure 3). All microfluidic experiments were performed within 6 hours of the initial blood draw.

To detect ATP release by cells, a solution of 450 μL Luciferin/Luciferase and 50 μL packed RBCs was prepared and loaded into a 1‐mL plastic syringe. The syringe was then connected to a syringe pump (Harvard Apparatus PHD 2000), and polyethylene tubing (inner diameter 0.58 mm) was used to connect the syringe to the inlet of the channel. RBCs were pumped through the channels at 0.25, 2.5, or 5 μL/min. A representative image of RBCs at 10% Hct flowing through the constriction at 2.5 μL/min is shown in Figure 1B. To prevent settling of RBCs, the syringe was rotated by hand every 30 seconds and was replaced after each individual trial.

To determine ATP concentration as a function of downstream position, *x*, we controlled a motorized stage using open source software from μManager. Here, *x *=* *0 corresponds to the end of the tapered region, and the beginning of the constriction (cf. Figure 1). The channel was typically scanned from *x *=* *−750 μm to *x *=* *6750 μm in steps of 250 μm, pausing for 10 seconds at each location. Light from the reaction between ATP and Luciferin/Luciferase was collected through a 63× magnification objective on an optical microscope (Leica DMI 3000 B), using a photomultiplier tube (Hamamatsu, model R1527P) installed in a Photon Technology International 814 system with a high voltage supply for signal amplification. The voltage output of the photomultiplier tube was collected using custom methods in LabVIEW and converted to PPS using in‐house algorithms written in MATLAB. The PPS values were then converted to ATP concentration using the previously obtained calibration curve (cf. Figure 2). Blood from each of the 15 participants was tested in triplicate at each of the three distinct flow rates (0.25, 2.5, or 5.0 μL/min), yielding 135 experimental trials in total.

## 3 RESULTS

Representative plots of the ATP concentration vs position downstream from the constriction for RBCs drawn from three different individuals, all flowing at 2.5 μL/min, are shown in Figure 3A. Discrete points represent the measured concentration, while solid lines represent a five‐point moving average. We emphasize that the RBCs from all three participants show evidence of mechanotransductive ATP release, as evidenced by the measurable ATP concentrations well above the threshold. However, two of the representative trials indicate no impact of the constriction on the ATP concentration; in other words, the ATP concentration remained approximately the same regardless of downstream position, at least over the field of view examined here. In contrast, one of the trials yielded a spike in ATP concentration similar in shape, to those observed by Wan et al.^12^

Inspection of all 45 trials at the flow rate 2.5 μL/min shows that, while all RBCs from all participants exhibited mechanotransductive release of ATP, a distinct spike in ATP concentration following the constriction was a rare occurrence (Figure 3B). The vast majority of trials yielded no discernible spike. For comparison, the results of Wan et al^12^ and Cinar et al^13^ are also superimposed in Figure 3B, highlighting the stark contrast. Notably, participants in our study show ATP concentrations below 0.6 μmol/L, that is, approximately 65% and 25% less than the peak concentrations observed by Wan et al^12^ and Cinar et al,^13^ respectively. More importantly, RBCs from only two of our 15 participants showed reproducible evidence of a mechanotransductive spike for all three trial replicates at this flow rate, while, RBCs from the remaining participants yielded only spatially invariant ATP concentrations. This behavior contrasts strongly with the results of Wan et al,^12^ which show a marked increase in ATP downstream of the constriction from 0.44 ± 0.05 to 1.57 ± 0.17 μmol/L. The magnitude of the error in comparison with the magnitude of the peak suggests this result was consistent for all trials conducted by Wan et al^12^ (n = 5). Similarly, the RBCs tested by Cinar et al^13^ show on average a higher concentration than our results and a very slight increase in ATP from 0.67 ± 0.12 to 0.81 ± 0.17 μmol/L. Note that the magnitude of the error for Cinar et al^13^ is on the order of the peak magnitude, complicating interpretation of whether a mechanotransductive spike occurred.

**Figure 4:**
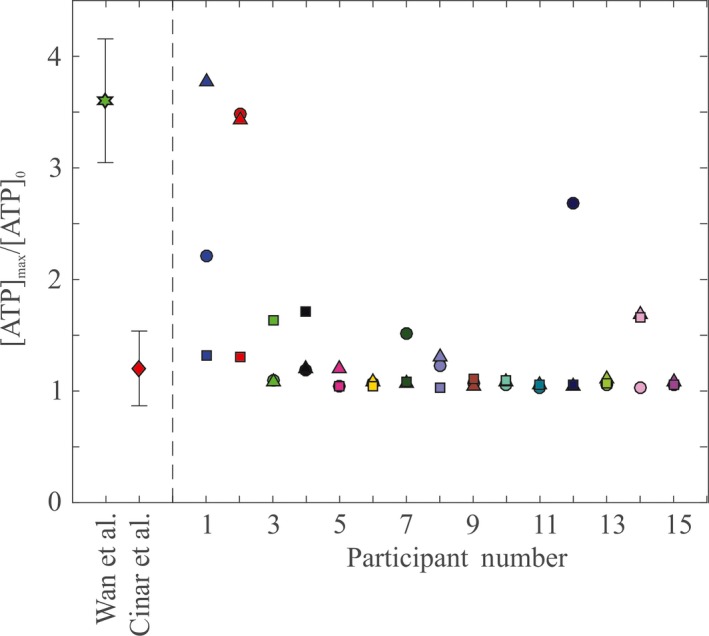
Ratio of peak to initial ATP concentrations. Shown to the right of the dashed line are values from this work for a flow rate of 2.5 μL/min. Different colors correspond to individual participants, while shapes denote separate trials for a single participant. To the left of the dashed line are previously reported results. The green star represents work by Wan et al,^12^ with error bars showing one standard deviation of n = 5 measurements for one participant. The red diamond represents work by Cinar et al,^13^ with error bars corresponding to the standard deviation of n = 11 measurements from 6 participants. For measurements taken in this work, the initial concentration of ATP is calculated as the average of all measurements taken before the constriction. For previous work, initial concentration of ATP is taken as the first measurement (cf. Figure 3)

To highlight differences in mechanotransductive behavior amongst all blood tested to date, Figure 4 compares the ratio of the maximum concentration of ATP observed, [ATP]_max_, to the initial concentration of ATP prior to the constriction, [ATP]_0_, for each participant. Note that this is a conservative definition for the spike magnitude because it does not depend on where the max value occurs, that is, it includes “peaks” that might instead more accurately be described as the consequence of random fluctuations in the measurements. Nonetheless, 12 participants in our study show [ATP]_max_/[ATP]_0_ between 1 and 1.7 for all trials in Figure 4, which is broadly consistent with the previous results of Cinar et al^13^ (1.2 ± 0.3). Participants with evidence of a mechanotransductive spike in all trials (n = 2) exhibit ATP ratios in the range of 1.5‐4, which is comparable to earlier work by Wan et al^12^ (3.6 ± 0.6). A single participant in this work showed evidence of a large peak for one trial ([ATP]_max_/[ATP]_0_ = 2.6) and ratios of ~1 for the other two trials. The high ratio (2.6) for this one trial is due to extremely low initial concentrations, [ATP]_0_ < 0.025 μmol/L, while the maximum concentration is only 0.05 μmol/L. For comparison, the initial concentrations ([ATP]_0_) of this participant's other two trials were approximately 0.1 and 0.2 μmol/L.

**Figure 5:**
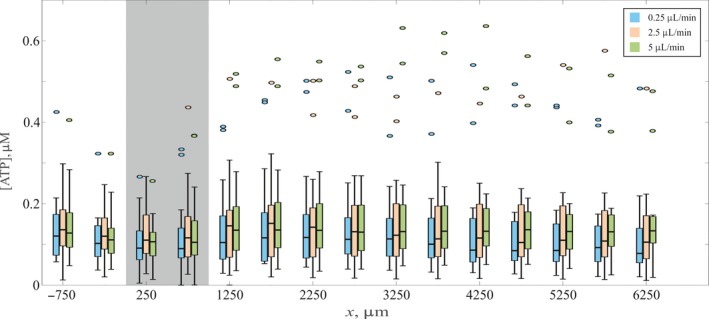
Effect of flow rate on deformation‐induced release of ATP by RBCs. Boxplots for the concentration of ATP at individual downstream positions are shown for 0.25 (blue), 2.5 (orange), and 5 μL/min (green). Colored boxes represent the span of the 25th to 75th percentile; black lines inside the boxes represent the median; and whiskers correspond to the maximum and minimum for each position. Points above each boxplot represent statistical outliers; these points represent the same participants who display evidence of a mechanotransductive spike (cf. Figures 2, 3, and 4). The gray shaded region on the figure corresponds to the constricted region of the channel. The boxes for different flow rates are slightly offset horizontally at each position for clarity

Figures 3 and 4 focused on our results using a flow rate of 2.5 μL/min. We also performed trials at smaller and larger flow rates, the results of which are summarized as box plots in Figure 5. Each box represents the quartiles and medians of a sample size of 45 ATP concentrations measured at each location along the channel. On average, there is a slight increase in the median value of ATP concentration for a given *x* position as the flow rate increases; given the scatter in the data, however, the increase is not statistically significant. The key feature is that, for each flow rate, the average detected ATP remains relatively constant along the entire length of the microfluidic channel and is between 0 and 0.8 μmol/L for all flow rates. In other words, changes in the applied shear stress within the constriction due to the increased flow rate yielded, on average, no significant changes from the baseline level of ATP prior to the constriction, at least over the range of flow rates explored here. The only exceptions are the outliers, defined here as [ATP] > *q*_3_ + 1.5 × (*q*_3_ − *q*_1_), where *q*_3_ is the 75th percentile (third quartile) and *q*_1_ is the 25th percentile (first quartile). Outliers are plotted as individual points above the main boxes in Figure 5. The RBCs from this minority of participants yielded an observable spike in ATP concentration following the constriction region (as typified in Figure 3A).

**Figure 6:**
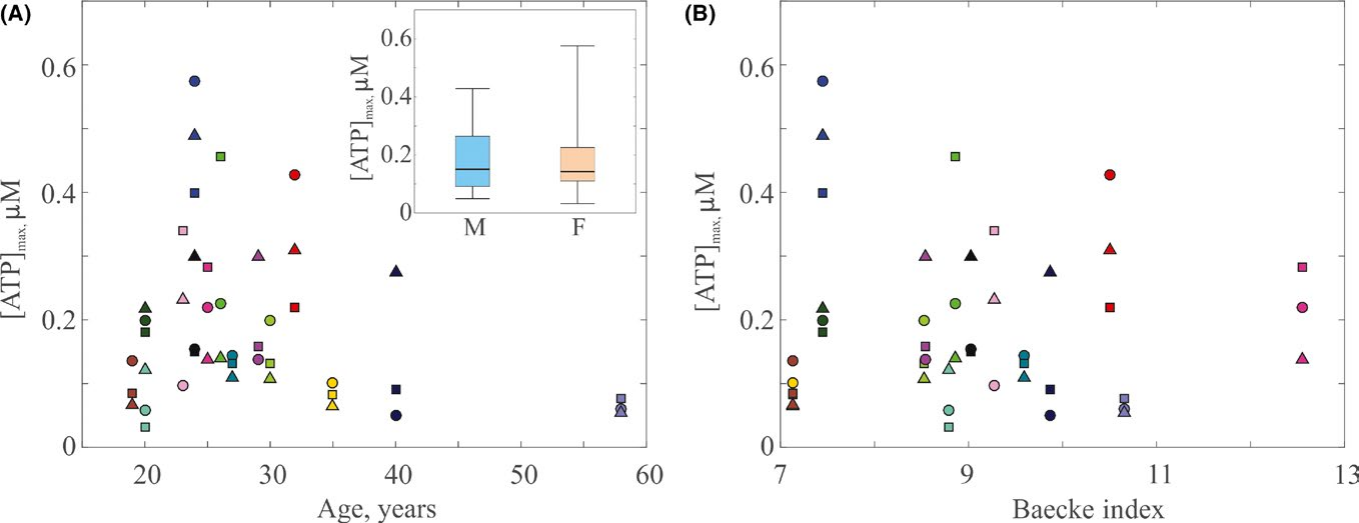
(A) Effect of age on deformation‐induced release of ATP (*R* = −0.25, *P* = .10). Inset shows the effect of gender on [ATP]_max_. Colored boxes represent the span of the 25th to 75th percentile; black lines inside the boxes represent the median; and whiskers correspond to the maximum and minimum. There is no statistically significant difference between the two sets (*P* = .40) for n_m_ = 9 and n_f_ = 6. (B) Effect of physical activity, as measured by the BI, on deformation‐induced release of ATP (*R* = −0.02, *P* = .87). Each point shows a single measurement of maximum detected ATP concentration at *Q* = 2.5 μL/min. Different colors correspond to different individual participants, while different shapes of the same color denote individual trials for a single participant

As the experimental results suggest that RBCs from some participants yield an appreciable mechanotransductive spike, while most do not, a natural question is as follows: what makes these participants different? Information about each participant is listed in Table S1. Here, we probe whether age, gender, or physical activity index affect ATP release. We compare [ATP]_max_ vs age for *Q* = 2.5 μL/min in Figure 6A. There is no significant evidence that increasing age corresponds to decreased ATP release by RBCs (*R* = −0.25, *P* = .10) for participants in this work, which is in disagreement with previous work by Kirby et al.^30^ However, the majority of our participants are between the ages of 20‐35, and only two participants are above the age of 40.

We analyzed differences in magnitude of ATP released by males and females. A comparison of the two is shown as an inset in Figure 6A. There are two boxplots representing the distribution of [ATP]_max_ for male (blue) and female (red) participants at a flow rate of *Q* = 2.5 μL/min. We find no statistically significant differences (*P* = .40) in detected ATP for n_m_ = 9 male and n_f_ = 6 female participants.

Finally, to probe the effect of physical activity level on ATP release by RBCs, we used the BI as a measure of physical activity. BI was determined using the Modified Baecke Questionnaire.^31^, ^32^ We compare [ATP]_max_ vs BI for *Q* = 2.5 μL/min in Figure 6B, which shows a lack of correlation between physical activity and ATP release by RBCs for the range of BIs explored here (*R* = −0.02, *P* = .87).

## 4 DISCUSSION

The major finding in this work is that for n = 15 participants, only two show evidence of a distinct mechanotransductive spike qualitatively consistent with the work of Wan et al.^12^ Previous work by Cinar et al^13^ shows a peak comparable to their reported error, making it unclear if a mechanotransductive spike had occurred. Additionally, there are differences in the reported timescales for mechanotransductive release between these three studies.

Wan et al^12^ reported that their mechanotransductive spike occurred at around *t *≈* *100 ms, where *t* is the time that has transpired from the entrance of the constriction,

(1) $$t=\frac{h}{Q}\left(w_{c}l_{c}+w_{w}\left(x-l_{c}\right)\right),$$

where *Q *=* *3 μL/min is the flow rate, *h *=* *37 μm is the height of the microfluidic channel, *w*_*c*_ = 20 μm is the width of the constriction region, *l*_*c*_ = 800 μm is the length of the constriction region, *w*_*w*_ = 100 μm is the width of the wide region, and *x* (in μm) is the distance travelled from the constriction. Solving for *x*, Wan et al^12^ found their peak at *x *≈* *1800 μm. For Cinar et al,^13^ all parameters remain the same but *h *=* *30 μm; however, the peak is found much further along the constriction at *t *≈* *150 ms or *x *≈* *3300 μm. Five of the peaks we observe in this work occur at *x *≈* *2750 μm, the remainder occur near the end of our microfluidic channel (*x *>* *6000 μm).

The main difference between our work and that of Wan et al^12^ and Cinar et al^13^ is the number of participants studied. In Wan et al,^12^ a single participant was used, while Cinar et al^13^ used 6 and this work used 15; thus detailed data now exist for 22 individuals. The observed differences in both the magnitude and the timescale of mechanotransductive response between studies suggest that the dynamics of ATP release by RBCs are much more complicated than originally believed. It appears that the mechanotransductive spike behavior initially described by Wan et al^12^ is limited to a very small subset of the population.

To estimate the size of this subpopulation, for the sake of specificity, we restrict attention to ATP spikes such that the average of an individual participant's trials yielded a ratio of peak to initial ATP concentrations that exceed a factor of two, that is,

(2) $$\frac{{\left[\text{ATP}\right]}_{\max}}{{\left[\text{ATP}\right]}_{\mathrm{init}}}\geq 2$$

We note this definition is quite conservative compared to the ratio of ~4 originally observed by Wan et al (cf. Figure 3). Using this definition, only 3 of 22 = 14% of participants tested to date have exhibited a spike. Given the small sample size, however, it is important to recognize that the true subpopulation size could appreciably differ from this estimate. To calculate a rigorous confidence interval for the proportion of individuals who exhibit a mechanotransductive spike, we use the Wilson score index as recommended by Brown et al^33^ for interval estimation of the probability of success in a small sample size binomial distribution (treating a spike as a “success” and no spike as a “failure”). The Wilson score confidence interval limits are

(3) $$w_{-},\;w_{+}=\frac{\hat{p}+\frac{z_{\alpha /2}^{2}}{2n}\pm z_{\alpha /2}\sqrt{\frac{\hat{p}\left(1-\hat{p}\right)}{n}+\frac{\left(z_{\alpha /2}^{2}\right)}{4n^{2}}}}{1+\frac{z_{\alpha /2}^{2}}{n}},$$

where $\hat{p}$ = n_*s*_/n, and *z*_α*/2*_ = 1.96 when α = 0.05 (95% confidence interval). With n_s_ = 3 and n* *=* *22, the interval limits are *w*_*−*_ = 0.05 and *w*_*+*_ **= 0.33. In other words, to 95% confidence, somewhere between 5% and 33% of the population is expected to have RBCs that yield a mechanotransductive spike as defined by Equation 2.

Another key feature of our results is that, while a slight increase in the median ATP concentration is seen when the flow rate was increased by a factor of 20 from 0.25 to 5 μL/min, it is not statistically significant. This observation broadly agrees with expectations based on previous work; several groups have found that increasing either the flow rate^34^ or decreasing the channel dimensions^9^, ^10^ yields higher ATP levels, although none tested the channel geometry used here. We would like to note that previous investigations of flow rate effects by Sprague et al^34^ utilized flow rates two orders of magnitude higher than those used here. Additionally, it is well known that a change in vessel dimensions has a significantly higher impact on the magnitude of the shear stress than a change in flow rate. The most direct comparison for us to make again is the microfluidic work by Wan et al,^12^ who found that as the constriction width decreased from 50 to 20 μm, the peak ATP concentration increased by a factor of ~5. The base levels of ATP release in the work of Wan et al,^12^ that is, before and after the spike, remained largely unaffected by the dramatic change in shear. Here, we alter shear stress not by changing the geometry but by increasing the magnitude of the flow from 0.25 to 5 μL/min. At each flow rate, the same two participants display evidence of a mechanotransductive spike (cf. outliers in Figure 5), although the magnitude of this spike was insensitive to increased flow rates within the range examined here. The lack of any observable impact on the ATP spike in this work differs from the earlier work by Wan et al,^12^ but the lack of a statistically significant impact on baseline levels of ATP release is similar.

In this work, the participants involved represented a variety of ages and physical activity backgrounds. While here we see no evidence that age affects magnitude of released ATP, work by Kirby et al^30^ suggests a negative correlation between age and ATP release. Expectations for dependence of ATP concentration on physical activity and gender are less clear; however, several groups have investigated effects of both factors on RBC deformability, which may affect the magnitude of ATP release.^9^, ^12^ There is evidence that long‐term aerobic training improves RBC deformability,^35^, ^36^ indicating higher physical activity levels may lead to increased ATP release. Work investigating the effect of gender offers competing conclusions, Guillet et al^37^ have suggested RBCs taken from female participants are more rigid, while work by Kameneva et al^38^ indicated increased rigidity in RBCs taken from male participants. It is anticipated that gender does not affect the magnitude of ATP released by RBCs, which agrees with the results found here. We emphasize that our study is relatively limited in that participants represent a narrow range of physical activity levels, and only two participants over the age of 40 are included. We recommend future studies explore concentrations of ATP released by a more inclusive age range, as well as populations of more highly trained athletes in comparison with their sedentary counterparts.

An important consideration in work investigating ATP release by RBCs is to determine whether the presence of ATP is due to mechanotransduction or hemolysis. Researchers have employed a variety of indirect techniques for measurement of lysis in microfluidic channels including direct observation of cells by high‐speed video,^12^ and comparisons of ATP release between healthy and rigidified cells.^5^, ^9^, ^10^, ^11^ Here, we follow the methodology of Wan et al^12^ and employ direct observation of cells from the previous high‐speed video work of this same group.^29^ In direct observation of over a hundred cells at each flow rate, no cell lysis was observed. However, indirect measurements of hemolysis cannot fully eliminate the possibility that cell lysis is a contributor to ATP release as was previously proposed in the work of Sikora et al^24^ Thus, one alternative explanation for the observed spike in mechanotransductive ATP release is that as cells move through the tapered and constriction regions of the channel a number of cells are lysed, which leads to the significant increase from baseline ATP measurements. Direct corroboration of this hypothesis by, for example, spectrophotometric measurements of hemoglobin levels is challenging due to the small volumes of fluid passed through the microchannel.

Another consideration in this work is that the average ATP concentration appears slightly lower than previous results using the same system. As we use the same Hct, it is unlikely this is due to a difference in total number of RBCs. However, it is anticipated that increases to the chosen Hct would increase the detected concentration of ATP in the channel, consistent with earlier investigations.^7^, ^9^ It is also unlikely that the differences in detected ATP are due to differences in enzyme efficacy. As stated previously, we prepare a de novo calibration curve for each day of experiments to confirm enzyme activity.

Other possible explanations for the lack of ATP spikes observed here are worth considering. In our work, a tourniquet was used during blood collection, consistent with the methodology used by Wan et al^12^ and Cinar et al^13^ Some studies on ATP mechanotransduction^2^, ^20^, ^21^ avoided the use of a tourniquet, as it may subject erythrocytes to increased shear forces prior to transduction experiments. As it is unclear whether isolated erythrocytes can release ATP repeatedly following shear stress, one potential concern is exposure to increased shear prior to mechanotransduction experiments. The RBCs in our work released ATP in all trials, implying the capacity of the RBCs to release ATP was not significantly affected by the use of a tourniquet. Most importantly, the two most direct comparisons for the current study, Wan et al^12^ and Cinar et al,^13^ both employed the tourniquets in their work. Thus, it is unlikely that the lack of an observable ATP spike for 13 of our 15 participants is due to premature release of ATP by RBCs during collection. Another possible explanation is that the work described by Wan et al^12^ is an artifact of an artificially low signal measured upstream and downstream of the constriction. However, Wan et al^12^ reproducibly observed large spikes in mechanotransductive ATP release across 65 experimental trials (albeit in blood drawn from a single donor). The magnitude of this spike was sensitive to both the magnitude and duration of shear stress in a consistent manner, complicating any potential interpretation in terms of a light calibration artifact. A simpler explanation is that most people's blood does not respond in a similar fashion.

A final interesting question, not addressed in the current work, is whether the mechanotransductive spikes are correlated with increased Ca^2+^ concentrations. As shown in the work of Cinar et al,^13^ there is evidence that Ca^2+^ influx may be important in the ATP release mechanism. Future work imaging the intracellular Ca^2+^ concentration as a function of position may provide further understanding of the underlying differences in participants who display a mechanotransductive spike and those who do not. These future studies would also benefit from additional tests to see whether lack of a mechanotransductive spike is correlated with lack of the ability to transduct ATP in response to other physiological stimuli such as hypoxia or changes in pH.

## PERSPECTIVE

Combining this study with the previous work by Wan et al^12^ and Cinar et al^13^ suggests that, while shear‐induced ATP release occurs with all donors, a mechanotransductive spike in ATP release by RBCs in response to increased shear stress occurs in approximately 14% of the population. It remains unclear what factors may contribute to these distinct mechanotransductive dynamics exhibited by blood from different individuals. Future work should examine a wider range of ages, flow rates, levels of physical activity, and also examine correlations with other parameters like Hct, Hb, or Ca^2+^ concentrations, to more completely understand their effect on the dynamics of deformation‐induced ATP release.
