# Supplementary material for: A spike in mechanotransductive adenosine triphosphate release from red blood cells in microfluidic constrictions only occurs with rare donors
Source: Microcirculation. 2018 Apr 11;25(3):e12439. doi: 10.1111/micc.12439 (PMC5947537; doi:10.1111/micc.12439)
Supplement: Supplementary file 1 [file MICC-25-na-s001.docx]

**Supporting information for****:**

**A spike in mechanotransductive adenosine triphosphate release from red blood cells in microfluidic constrictions only occurs with rare donors**

J. E. Mancuso and W. D. Ristenpart

Department of Chemical Engineering, University of California Davis, Davis, CA 95616, USA.

| Participant | Age | Gender | Baecke Index |
| --- | --- | --- | --- |
| 1 | 24 | f | 7.45 |
| 2 | 32 | m | 10.51 |
| 3 | 26 | f | 8.86 |
| 4 | 24 | m | 9.02 |
| 5 | 25 | m | 12.55 |
| 6 | 35 | m | 7.13 |
| 7 | 20 | f | 7.45 |
| 8 | 58 | m | 10.65 |
| 9 | 19 | f | 7.13 |
| 10 | 20 | f | 8.78 |
| 11 | 27 | f | 9.60 |
| 12 | 40 | m | 9.87 |
| 13 | 30 | m | 8.53 |
| 14 | 23 | m | 9.28 |
| 15 | 29 | m | 8.54 |

Table S1: Collected information about participants
